# A three-dimensional collagen scaffold cell culture system for screening anti-glioma therapeutics

**DOI:** 10.18632/oncotarget.10885

**Published:** 2016-07-28

**Authors:** Donglai Lv, Shi-cang Yu, Yi-fang Ping, Haibo Wu, Xilong Zhao, Huarong Zhang, Youhong Cui, Bing Chen, Xia Zhang, Jianwu Dai, Xiu-wu Bian, Xiao-hong Yao

**Affiliations:** ^1^ Institute of Pathology and Southwest Cancer Center, Southwest Hospital, Third Military Medical University, Chongqing, China; ^2^ Key Laboratory of Tumor Immunopathology, Ministry of Education of China, Chongqing, China; ^3^ State Key Laboratory of Trauma, Burns and Combined Injury, Institute of Combined Injury, School of Military Preventive Medicine, Third Military Medical University, Chongqing, China; ^4^ Institute of Genetics and Development, Chinese Academy of Sciences, Beijing, China

**Keywords:** chemosensitivity, collagen scaffold, glioma stem cells, three-dimensional culture, MGMT

## Abstract

Three-dimensional (3D) culture, which can simulate *in vivo* microenvironments, has been increasingly used to study tumor cell biology. Since most preclinical anti-glioma drug tests still rely on conventional 2D cell culture, we established a collagen scaffold for 3D glioma cell culture. Glioma cells cultured on these 3D scaffolds showed greater degree of dedifferentiation and quiescence than cells in 2D culture. 3D-cultured cells also exhibited enhanced resistance to chemotherapeutic alkylating agents, with a much higher proportion of glioma stem cells and upregulation of O6-methylguanine DNA methyltransferase (MGMT). Importantly, tumor cells in 3D culture showed chemotherapy resistance patterns similar to those observed in glioma patients. Our results suggest that 3D collagen scaffolds are promising *in vitro* research platforms for screening new anti-glioma therapeutics.

## INTRODUCTION

Malignant glioma is the most common and deadly type of brain tumor [1]. In the past decade, even with improvements in surgical, radiation and chemotherapeutic methods to treat glioblastoma multiform (GBM), the most malignant glioma (World Health Organization [WHO] grade IV), the median patient survival has only increased from 10 months to 14 months [2]. New, more effective treatment regimens are urgently needed. Most drugs failed to achieve satisfactory effects in a number of recent multi-center anti-glioma Phase II clinical trials. It has been shown that traditional two-dimensional (2D) cell culture systems perform poorly as preclinical drug discovery tools, and are not suitable models for investigating solid tumors [3–5]. Current 2D cell culture systems provide neither good glioma stem cell (GSC) enrichment nor biomimetic microenvironments, including appropriate architecture, extracellular matrix (ECM) components and cell interactions [6]. This leads to large deviations in drug sensitivities between *in vitro* tests and *in vivo* clinical evaluations [3]. Therefore, building new *in vitro* anti-glioma drug research models will be crucial for the development of effective anti-glioma therapeutics [7].

To address these challenges, several 3D tumor cell culture techniques have been reported [8–11]. Cancer cells cultured in 3D structures may be superior for use in *in vitro* trials due in part to increased cell-cell and cell-ECM interaction. 3D scaffolds may better simulate native tumor microenvironment ECM [12] and provide more accurate drug efficacy analyses [13]. The principal ECM component identified in the normal brain is hyaluronan (HA) [14], therefore brain tissue engineering studies, including those for malignant tumors [15], frequently choose HA as a matrix-mimetic platform. However, glioma ECM composition is critically different from that of normal brain. Glioma tissues contain large amounts of fibrillary collagens [16], which are important ligands for activation of signal transduction networks required for glioma malignancy [17]. In this study, we proposed that collagen is a superior biomaterial for *in vitro* glioma studies. We developed a porous collagen scaffold and constructed a 3D glioma culture model using this scaffold.

To evaluate anti-glioma drug efficacies and to clarify different drug-resistance mechanisms, we performed *in vitro* trials using our 3D collagen scaffolds. Morphology, proliferation, growth kinetics, and chemosensitivity of glioma cells in 3D collagen scaffolds were remarkably different from their 2D monolayer counterparts. Relatively slow cell growth in the 3D model was attributed to decreased proliferation and increased quiescence. Dedifferentiation and increased drug resistance were also observed in 3D-cultured glioma cells. Drug resistance was attributed to MGMT upregulation and enhanced glioma cell stemness.

## RESULTS

### Morphology and structure of glioma cells in 3D culture

**Figure 1:**
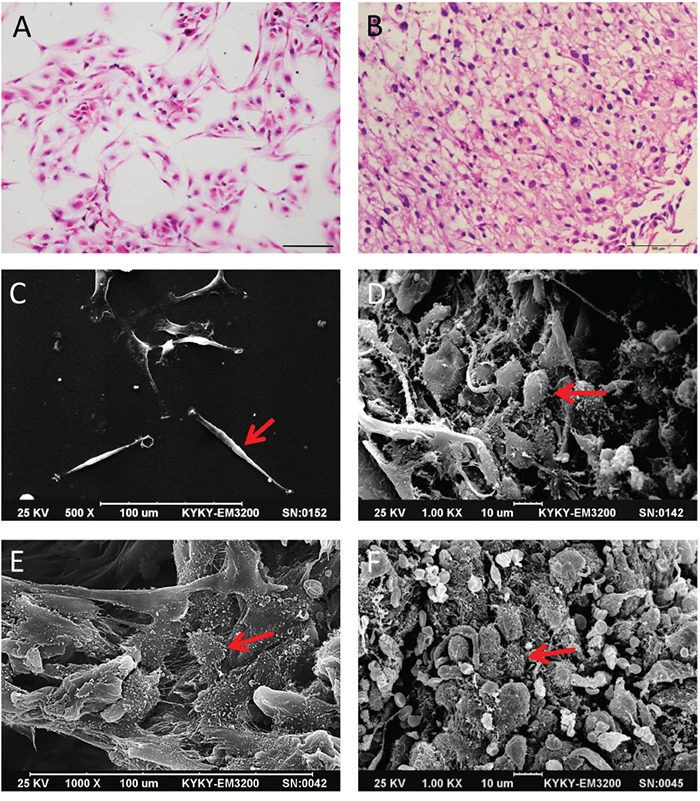
Comparison of glioma cell morphology by H&E staining and SEM Primary glioma cells in 2D and 3D culture with H&E staining **A** and **B.** Scale bar = 100 μm. U87 cells in 2D and 3D culture in SEM image **C** and **D.** Scale bars = 100 μm and 10 μm. Primary glioma cells in 3D scaffolds and human glioma tissue imaged by SEM **E** and **F.** Scale bars = 100 μm and 10 μm. Red arrow indicates glioma cells.

We observed changes in cell morphology in 3D collagen scaffold cultures as compared to 2D cultures. After seven days in culture, U87 and primary glioma cells were fixed, dehydrated and embedded in paraffin for H&E staining or dried for SEM imaging. Glioma cells in 3D collagen scaffolds (Figure 1B) but not in 2D culture plates (Figure 1A) displayed a high degree of similarity with primary tumor tissue. SEM showed that U87 cells in 2D culture were fusiform, flat and epithelioid (Figure 1C). Glioma cells in 3D scaffolds grew as small, round or ovoid cells appeared stereoscopic and formed a multi-layer structure (Figure 1D). Primary tumor cells cultured in 3D collagen scaffolds (Figure 1E) were morphologically similar to glioma cells in human tumor tissues (Figure 1F), and grew in complex formations with cilia or microvilli on their surface. Furthermore, with increased culture duration (3 to 10 days), cells constituted 3D structures throughout the deep scaffold (Supplementary Figure S1A–S1D). These results suggest that 3D collagen scaffolds more effectively mimic the *in vivo* microenvironment than 2D cultures.

### Growth profile of glioma cells in 3D culture

**Figure 2:**
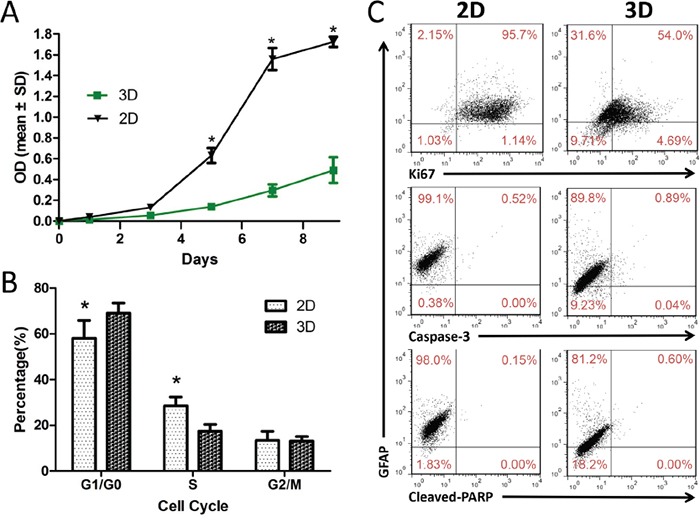
U87 cell proliferation and dedifferentiation in 3D collagen scaffolds U87 cell proliferation in 2D and 3D culture assessed at different time points **A.** 3D culture induces accumulation of cells in G0/G1 phase with concomitant reduction of cells in S phase **B.** Levels of Ki67, caspase-3, cleaved-PARP and GFAP in U87 cells in 2D and 3D culture as measured using flow cytometry **C.** Results are shown as the means ± SD. *P<0.05.

We compared proliferation and cell cycle stage in glioma cells cultured in 3D collagen scaffolds with cells in 2D monolayer cultures. CCK8 assay results showed that U87 cells grew more slowly in 3D scaffolds than in 2D monolayer cultures (Figure 2A). Statistically significant differences were observed after five days in culture. As compared to 2D culture, in 3D culture the proportion of cells in G1/G0 phase increased from 58.05 ± 7.76% to 69.37 ± 4.20%, and cells in S and G2/M phases decreased from 28.51 ± 3.85% to 17.45 ± 3.02% and 13.44 ± 3.96% to 13.18 ± 1.82%, respectively (Figure 2B). This suggests that cells grown in 3D scaffold culture accumulated in G0/G1 phase with concomitant reduction in S phase. We also used flow cytometry to determine whether 3D culture altered U87 cell proliferation, apoptosis and differentiation. The proportion of Ki-67^+^, caspase-3^+^ and cleaved PARP^+^ U87 cells was 58.69%, 0.93% and 0.60%, respectively, in 3D culture and 96.84%, 0.52% and 0.15%, respectively, in 2D culture. On the other hand, the mean proportion of GFAP^+^ U87 cells was 98.31 ± 1.01% in 2D monolayers versus 86.03 ± 3.64% in 3D scaffolds (Figure 2C). A similar effect was seen on primary glioma cells (Supplementary Figure S2). The results showed that 3D culture induced glioma cell dedifferentiation and decreased proliferation but did not impact apoptosis. As determined by flow cytometry, slower cell growth in 3D scaffolds could be attributed to both decreased proliferation and increased quiescence. Similar apoptosis rates between 2D and 3D cultures indicate that our collagen scaffolds exhibit good biocompatibility.

### Response to chemotherapeutic drugs

**Figure 3:**
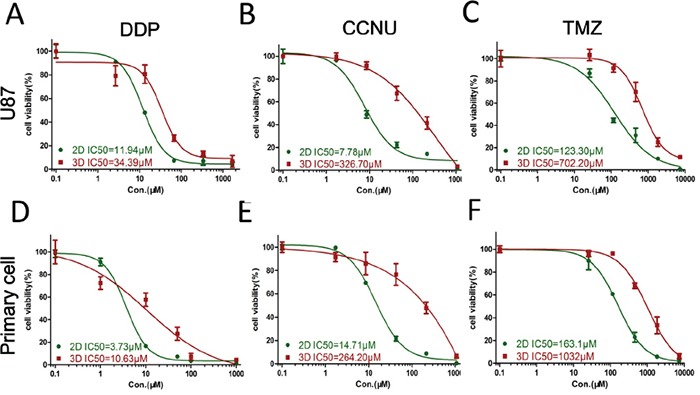
Glioma cell responses to chemotherapeutics U87 and primary glioma cell viabilities in 2D and 3D culture after exposure to DDP **A** and **D.** CCNU **B** and **E.** and TMZ **C** and **F.** Data represent the mean percentage viability (2D and 3D; left axis) ± SD normalized against untreated control cells.

DDP is the most commonly used cytotoxic chemotherapeutic agent, and CCNU and TMZ are the most common alkylating drugs clinical administered to glioma patients. U87 and primary glioma cells in 3D culture demonstrated greater resistance to all three drugs than cells in monolayer culture. Cells were more resistant to CCNU and TMZ than DDP. For U87 cells, the half-maximal inhibitory concentrations (IC_50_) in 3D and 2D cultures were: DDP: 34.39 and 11.94 μM, CCNU: 326.70 and 7.78 μM, TMZ: 702.20 and 123.30 μM, respectively (Figure 3A–3C). For primary glioma cells, IC_50_s in 3D vs. 2D cultures were: DDP: 10.63 and 3.73 μM; CCNU: 264.20 and 14.71 μM; TMZ: 1032.00 and 163.10 μM (Figure 3D–3F). These data suggest that glioma cells in 3D culturing are more chemotherapeutic resistant as compared with cells in 2D culturing.

### Changes in the expression of chemotherapy resistance-related genes

**Figure 4:**
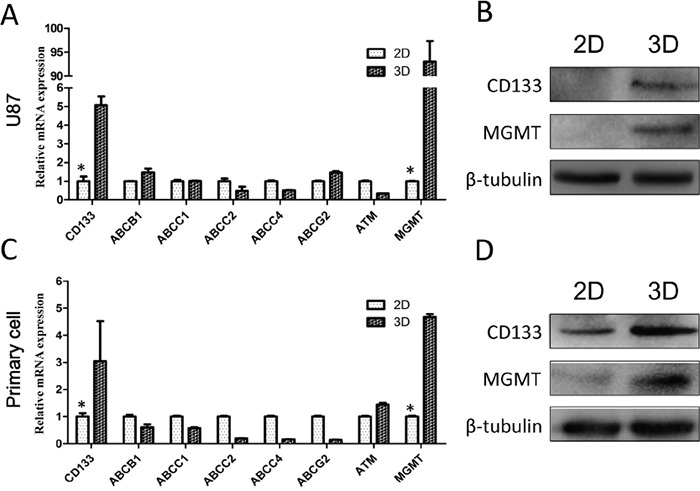
Chemotherapy resistance mechanisms shown by 3D-cultured glioma cells Levels of drug resistance-related genes were examined by qRT-PCR in U87 **A.** and primary glioma cells **C.** Expression data was normalized against GAPDH. MGMT and CD133 expression in U87 **B.** and primary **D.** cells via Western blotting. *P<0.05.

We investigated levels of multiple drug resistance-related genes in U87 and primary glioma cells, including genes related to drug efflux (ABCB1, ABCC1, ABCC2, ABCC4, ABCG2, ATM), DNA damage repair (MGMT) and stemness (CD133). We found that both CD133 and MGMT were upregulated in U87 and primary glioma cells in 3D culture (Figure 4A–4D). Importantly, U87 is MGMT-negative in traditional 2D culture [18]. These results suggest that drug resistance in cells grown in 3D collagen scaffolds could be attributed to increased DNA damage repair and stemness. The enhanced stemness phenotype agreed with the observed increases in the number of cells in G0/G1 phase and cellular dedifferentiation in 3D culture.

### Stemness in 3D-cultured glioma cells

**Figure 5:**
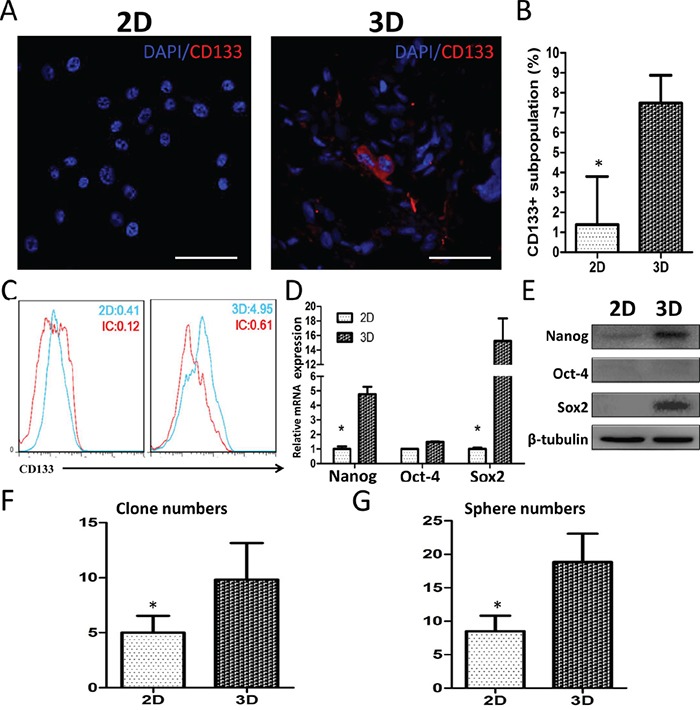
U87 cell stemness in 3D culture CD133 expression shown by confocal microscopy **A** and **B.** and flow cytometry **C.** Scale bar = 100 μm. Relative Nanog, Oct4 and Sox2 mRNA **D.** and protein **E.** levels in cultured cells as measured by qRT-PCR and Western blotting. qRT-PCR data was normalized against GAPDH. Colony and sphere formation by U87 cells in 3D culture **F** and **G.** Data represent the means ± SD. *P<0.05.

GSC-like properties were analyzed in U87 cells by immunofluorescent (IF) staining, FACS, qRT-PCR, Western blotting and colony/sphere-forming tests. CD133 IF staining was observed in 3D, but not 2D, culture slides (Figure 5A). The mean ratio of CD133^+^ cells to total cells was 1.39 ± 1.96% in 2D cultures vs. 7.48 ± 1.13% in 3D cultures (Figure 5B). Flow cytometry showed that 0.29% and 4.34% of GSCs were CD133^+^ in 2D and 3D cultures, respectively (Figure 5C). The proportion of GSC-like U87 cells grown in collagen scaffolds was 15-fold higher than that in monolayer culture. The stemness factors, Nanog and Sox2, were upregulated by 4.77 ± 0.51-fold and 15.25 ± 3.11-fold, respectively, in 3D cultured U87 cells (Figure 5D–5E). We also found that 3D culture increased U87 cell colony and spheroid formation (Figure 5F–5G) by approximately two-fold compared with cells in 2D culture. Enhanced stemness-associated properties were also observed in primary glioma cells in 3D culture (Supplementary Figure S3). These data indicate that 3D collagen scaffolds promote glioma cell stemness, activation of GSCs-associated factors and self-renewal.

### Chemotherapeutic drug treatments

**Figure 6:**
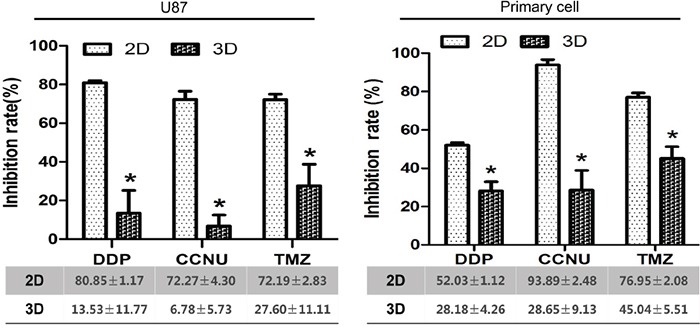
Inhibition of growth of U87 and primary glioma cells by DDP, CCNU and TMZ in 2D vs. 3D culture *P<0.05.

DDP, CCNU and TMZ are prototype chemotherapeutic agents verified in previous clinical trials. U87 and primary glioma cell inhibition efficacy was investigated according to individual drug peak plasma concentrations (PPCs) as measured in human blood. In 2D culture, the mean inhibition rates of DDP, CCNU and TMZ were 80.85 ± 1.17%, 72.27 ± 4.30% and 72.19 ± 2.83% in U87 cells, and 52.03 ± 1.12%, 93.89 ± 2.48% and 76.95 ± 2.08% in primary glioma cells. In 3D culture, the rates were 13.53 ± 11.77%, 6.78 ± 5.73% and 27.60 ± 11.11% in U87 cells, and 28.18 ± 4.26%, 28.65 ± 9.13% and 45.04 ± 5.51% in primary glioma cells (Figure 6). The chemosensitivity of glioma cells in 3D culture more closely resembled clinical objective response rates for all drugs as compared to 2D cultures.

## DISCUSSION

Due in part to the complexity of the tumor microenvironment, traditional 2D culture of tumor cells often does not accurately mimic the *in vivo* properties. Previous studies in 3D glioma culture systems commonly used gels, such as matrigel [19] or hydrogel [20]. Although these systems exhibit good biocompatibility, cell viability tests, such as MTT, CCK8 or Alamar Blue assay, are difficult to perform in gels. Thus, tumor studies in gels are usually focused on invasion, motility or specific signaling pathway activation. Compared with gel, the 3D scaffold system is suitable for cancer screening of therapeutics. A previous glioma drug study was performed using a chitosan-hyaluronic acid (HA) scaffold [15], which induced weak drug resistance corresponding to increased ABCG2 expression. However, MGMT overexpression is a main reason for glioma drug resistance in clinical cases [21], and collagen, not hyaluronic acid, is the main ECM component in glioma tissues. Use of appropriate ECM components is therefore critically important in monitoring tumor cell responses to exogenous cues such as growth factor activation or chemotherapy. In this study, we developed a porous 3D scaffold mainly containing collagens I and IV, the primary ECM components in glioma [22]. These scaffolds have several advantages, including high porosity, a large surface area-to-volume ratio for cellular attachment, tunable pore diameter, low cost and high reproducibility of culture condition. Using this scaffold, we created a 3D glioma culture model with features different from those of conventional *in vitro* 2D culture systems. Increased chemotherapy resistance was observed in 3D culture tumor cells as a result of increased stemness and upregulation of cell self-rehabilitation protein, MGMT. To our knowledge, this is the first observation that changing culture dimensions and ECM components induces MGMT overexpression by glioma cells.

The mechanism for enhancement of glioma cell stemness in 3D culture is not yet clear. GSC-enriched microenvironments are complex, but frequently include a variety of cytokine changes, hypoxia and poor local nutritional conditions [23]. 3D culture may provide regions of hypoxia and improve cell-cell communications [24, 25]. Hypoxia can reduce cell sensitivity to growth signals and cause the accumulation of GSCs in G0 phase of the cell cycle [26, 27], especially in 3D organoid systems [28]. This may explain increased number of quiescent cells in 3D scaffold cultures.

Drug resistance in glioma is associated with drug efflux, metabolism, cancer stem cells, DNA damage repair, and miRNA activity [29, 30]. Previous studies have described the relationship between cancer cell stemness and tumor chemosensitivity. For example, TMZ may fail to inhibit GSC self-renewal [31] via activation of the EGFR and SHH pathways [32] or various ATP binding cassette transporters [33]. Additionally, GSC quiescence and long life span also enhance drug resistance [34, 35]. Consistent with clinical results, we found that MGMT was upregulated in glioma cells in 3D collagen scaffold cultures. High MGMT activity in glioma promotes resistance to alkylating agent chemotherapeutics [36–38], as shown by increased resistance of our 3D-cultured glioma cells to CCNU and TMZ. Notably, U87 cells in 2D culture are MGMT-negative, but MGMT was upregulated in cells grown on the 3D scaffolds. Sox2 activation was also found in glioma cells in 3D culture, and MGMT activation may be induced by Sox2 [39]. Therefore, 3D collagen scaffold culture provides glioma cells with a microenvironment conducive to drug resistance, at least in part through GSC enrichment and enhanced DNA damage repair.

The reported clinical response rates of glioma patient to chemotherapeutics DDP, CCNU and TMZ were 10% [40], 12~21% [41, 42] and 7~40% [43–45], respectively. Our data suggest that treatment of glioma cells with these drugs in 3D culture more closely simulated clinical response rates as compared to cells in 2D culture.

In conclusion, we developed an *in vitro* 3D glioma cell culture system using collagen scaffolds that provide cell attachment structures. Glioma cells grown in these 3D cultures exhibited morphological and biochemical differences compared with cells grown in conventional 2D cultures, including enhanced chemotherapy resistance, GSC enrichment and MGMT overexpression. Importantly, tumor cells in 3D culture showed similar chemotherapy resistance patterns as those observed in glioma patients. Our results thus suggest that 3D collagen scaffolds are promising *in vitro* research platforms for screening new anti-glioma therapeutics.

## MATERIALS AND METHODS

### Preparation of 3D collagen scaffolds

Collagen scaffolds were fabricated from collagen membranes, made from bovine collagen of spongy bone, obtained from the Institute of Combined Injury of the Third Military Medical University. Briefly, collagen membranes were immersed in 0.5 M acetic acid for 8 h at 4°C, mixed in a blender for 15 min and neutralized by 4 M NaOH. The homogeneous solution was dialyzed in deionized water for 5 d and lyophilized. We produced collagen scaffolds with 50 μm average pore sizes. Scaffolds were cut into 1×5×5 mm pellets and cross-linked by 1 mg/ml 1-ethyl-3-(3-dimethyl aminopropyl) carbodiimide and 0.6 mg/ml N-hydroxysuccinimide. After crosslinking, pellets were lyophilized again, sterilized by 60Co and stored at ambient temperature.

### Primary glioma samples and cells

Tissue collection and analysis were approved by the Ethics Committee of Southwest Hospital, Third Military Medical University, and written informed consent was obtained from the participants. Human glioma tissues used for primary cell culture were obtained from one GBM patient who underwent neurosurgical operation in December 2009. The diagnosis was verified by pathological analysis and classified according to the WHO classification standard. Primary glioma cell isolation and culture was performed in our lab as described previously [46, 47]. The GBM cell line, U87, was purchased from the American Type Culture Collection.

### 2D and 3D glioma cell culture

For 2D culture, cells were seeded onto 60 mm dishes and maintained in DMEM supplemented with 10% FBS and 1% penicillin-streptomycin at 37°C and 5% CO_2_. For 3D scaffold culture, following immersion in 10% FBS-supplemented medium for 12h at 37°C, collagen scaffolds were loaded with cell suspensions (200,00 cells in 20 μL medium per scaffold) and maintained at 37°C for 4 h. Then, scaffolds with seeded cells were transferred to 6-well cell culture plates containing 3 ml medium; medium was changed every 2 days.

### Cell morphology analysis

Cell morphology was observed via Hematoxylin and eosin (H&E) staining and Scanning Electron Microscopy (SEM). Specimens (7 days) were fixed in 4% paraformaldehyde, embedded in paraffin, cut into for 5 μm sections and H&E stained. For SEM analysis, scaffold or glioma samples were fixed in 2.5% glutaraldehyde overnight, dehydrated in an ethanol gradient (70–100%), air-dried overnight, sputter-coated with gold and imaged (KYKY EM-3200). Glioma samples were quickly frozen in liquid nitrogen after surgical resection until use and fragmented immediately after removal from liquid nitrogen. A specimen with a diameter of about 1.5 mm was chosen for fixation.

### Cell proliferation assay

Cell viabilities in both 2D monolayers and 3D scaffolds were measured indirectly by the Cell Counting Kit-8 (CCK8) (Beyotime Technologies, C0038). Briefly, the original medium was replaced with 100 μl medium containing 10% CCK-8. The reaction was incubated at 37°C for 1.5 h and the solution was moved to a new 96-well plate for spectrophotometric measurement at 450 nm using a microplate reader (Varioskan Flash, Thermo Electron Corporation). Four parallel replicates of each sample were analyzed daily. DMEM containing 10% CCK-8 was used as a control.

### Flow cytometry

For cell cycle analysis, glioma cells (5 days) were washed twice with PBS and fixed in 75% cold ethanol at 4°C overnight. Fixed cells were washed with cooled PBS and stained using the cell cycle assay kit (Bestbio, BB-4104). The experiment was repeated three times. For proliferation and apoptosis analysis, Caspase-3, Ki-67 and cleaved poly (ADP-ribose) polymerase (PARP) were labeled with conjugated monoclonal antibodies (BD Biosciences) after U87 cell permeabilization. For CD133 expression analysis, cells were blocked by FcR blocking reagent (Miltenyi, 130-059-901) and then incubated with CD133/1-APC (Miltenyi, 130-090-826) at 4°C for 30 min. Cells were washed twice and resuspended with cold PBS. 7-AAD (BD Biosciences, 51-68981E) was added to cell suspensions to identify dead cells. Mouse IgG1-APC (Miltenyi, 130-092-214) was used as the isotype control. All analyses were performed on a FACS Calibur analyzer (BD Biosciences) using FlowJo software (Tree Star).

### Immunofluorescent staining

Samples were fixed overnight in 4% paraformaldehyde, cut into 15 μm sections and affixed to slides. Slides were blocked with 10% BSA for 1 h, incubated with anti-human CD133 (Boster, BA3992) overnight at 4°C, then washed three times in PBS and incubated with Alexa Fluor 647 secondary antibodies (Invitrogen, A21244) in the dark for 1 h at room temperature. Finally, slides were stained with 0.1% 4′, 6-diamidino-2-phenylindole (DAPI) to visualize cell nuclei, washed twice with PBS and examined via confocal microscopy (Leica LSM780). Image analysis was performed using ZEN software. Four random microscopy fields were selected for quantitative analysis, and the ratio of CD133^+^ cells to total cells in each field was determined by manual counting.

### Chemosensitivity assay

Dose–responses for chemotherapeutics were evaluated in 2D and 3D cultures. Glioma cells (5×10^3^ cells/well) were seeded on either monolayer or scaffold in a 96-well plate and allowed to grow for 24 h before treatment. Then, culture medium was replaced with fresh medium containing various concentrations of the anticancer drugs, temozolomide (TMZ) (Meilun, 85622-93-1), lomustine (CCNU) (Meilun, 13010-47-4) and cisplatin (DDP) (Sigma, 479306), for IC50 analysis. To better compare with clinical outcomes, anti-glioma inhibition rates were investigated according to drug PPCs of human blood in 2D and 3D culture. PPCs of the three tested drugs were TMZ: 258.0 μM, CCNU: 14.0 μM and DDP: 12.8 μM [48–50]. After 24 h, drug-containing medium was replaced with fresh medium and cells were incubated for 48 h. After treatment, chemosensitivity was determined using the CCK8 assay. Percent anti-glioma inhibition rates were calculated as the average cell viability in each drug group as compared to the average viability in the untreated group. All experiments were performed in triplicate.

### RNA isolation and quantitative RT-PCR

Total mRNA was isolated from tumor cells (5 day) using TRIzol™ Reagent (Invitrogen), following the manufacturer's instructions. cDNA was synthesized using the PrimeScript® First Strand cDNA Synthesis Kit (TaKaRa, D6110A). QRT-PCR was performed via the CFX96 Real-Time PCR Detection System (Bio-RAD,185-5195) with the SYBR® PrimeScript™ RT-PCR Kit (TaKaRa, DDR083A). Transcript levels were normalized to GAPDH.

### Western blotting

On day 5, cells cultured in 2D and 3D systems were washed twice in PBS and lysed using the M-PER® Mammalian Protein Extraction Reagent (Thermo, 75801) supplemented with proteinase inhibitor cocktail (Roche, 04693116001) for 30 min on ice. Whole-cell lysates were harvested from the supernatant by centrifugation at 12,000 g for 15 min. Lysates were electrophoresed in SDS-polyacrylamide gels (Beyotime Technologies, P0012A) and transferred to PVDF membranes (Millipore, IPVH00010). Primary antibodies used in this study included: anti-CD133 (Millipore, W6B3C1), anti-MGMT (Cell Signaling, 2739S), anti-Sox2 (Cell Signaling, 3579P), anti-Nanog (Cell Signaling, 4903), anti-Oct4 (Cell Signaling, 2750), anti-GAPDH (Bioworld, AP0063) and anti-β-tubulin (Cell Signaling, 2146S). A secondary HRP-linked goat anti-rabbit IgG antibody (Thermo, 31210) or goat anti-mouse IgG antibody (Thermo, 31431) was used as appropriate. Results were visualized using SuperSignal West Dura chemiluminescent substrate (Thermo Fisher Scientific).

### Colony and sphere formation assays

For colony formation assays, U87 cells from different culture models were plated (200 cells/well) in complete medium in a 24-well plate, and were allowed to form colonies for 12 days. Colonies were fixed with acetic acid and methanol (1:3) for 15 min and stained with 0.5% crystal violet for 30 min. Colonies containing more than 50 cells were counted manually. For sphere formation assays, cells were seeded in serum-free neural stem cell medium (DMEM/F12 medium containing 20 ng/ml recombinant human epidermal growth factor, 20 ng/ml basic fibroblast growth factor and 2% B27) (200 cells/well) into a 96-well plate. Spheroids were counted manually by inverted phase contrast microscopy after 10 days.

## SUPPLEMENTARY MATERIALS FIGURES


