# Supplementary material for: A three-dimensional collagen scaffold cell culture system for screening anti-glioma therapeutics
Source: Oncotarget. 2016 Jul 28;7(35):56904–14. doi: 10.18632/oncotarget.10885 (PMC5302961; doi:10.18632/oncotarget.10885)
Supplement: Supplementary file 1 [file oncotarget-07-56904-s001.pdf]

A three-dimensional collagen scaffold cell culture system for screening anti-glioma therapeutics

SUPPLEMENTARY FIGURES

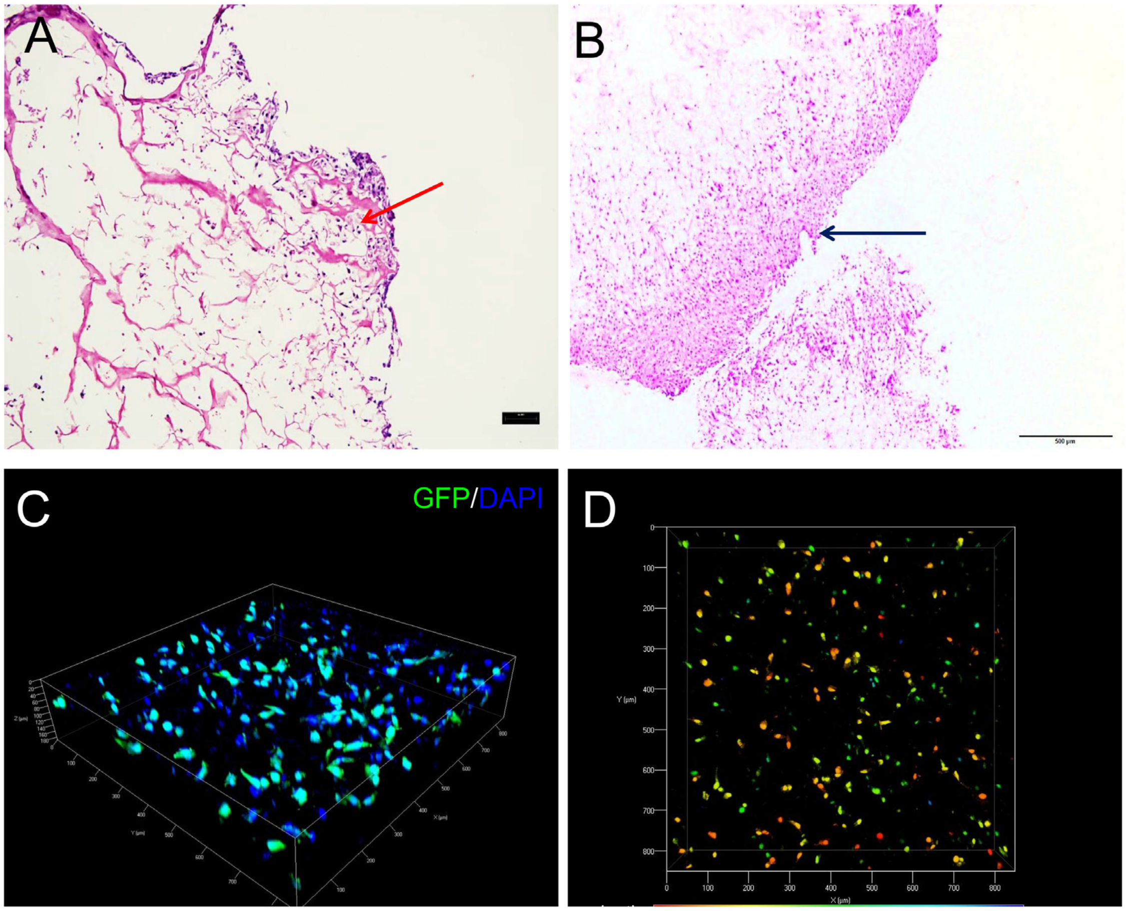

Supplementary Figure S1: U87 cell grown inside collagen scaffolds. H&E stained images of U87 cells penetrating collagen scaffolds were taken on days 3 **A.** and 10 **B.** Scale bars = 100 μm and 500 μm. Confocal microscopy indicated 3D distribution **C.** and nucleus depth **D.** of cells in collagen scaffolds.

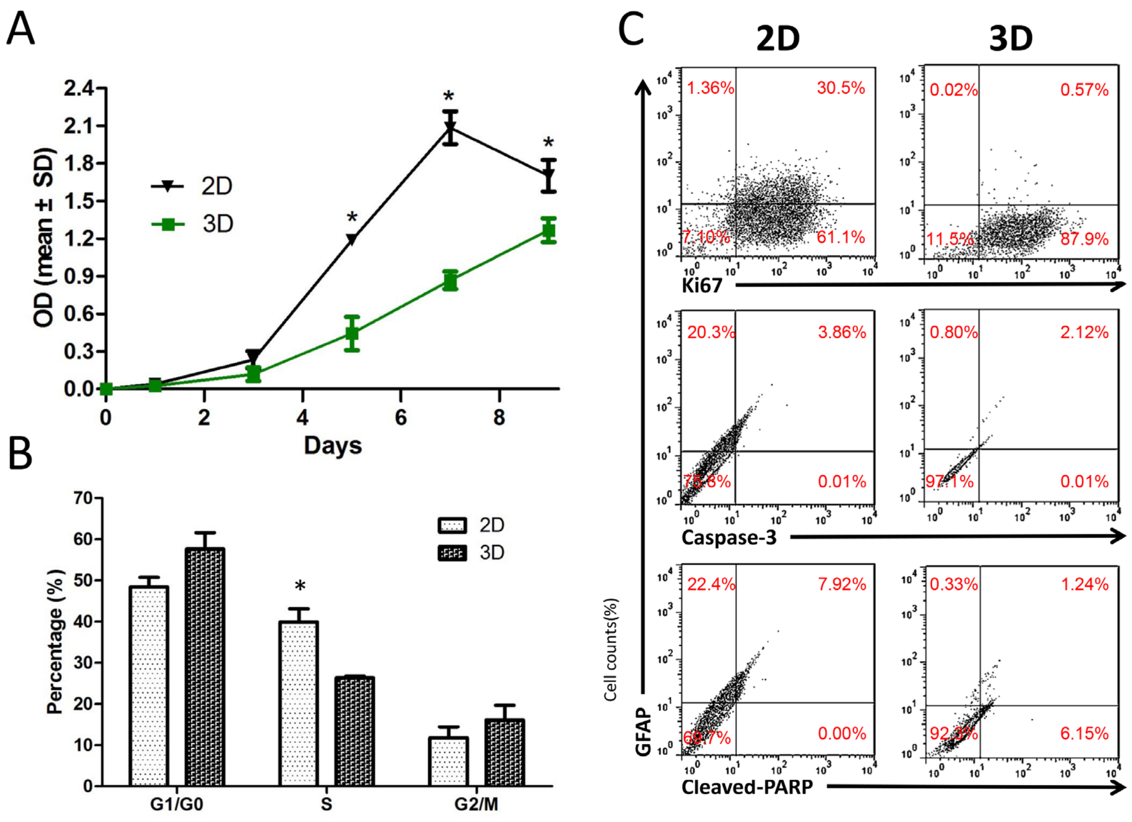

Supplementary Figure S2: Primary glioma cell proliferation and dedifferentiation in 3D collagen scaffolds. Primary glioma cell proliferation in 2D and 3D cultures as assessed at different time points **A.** 3D culture induces accumulation of cells in G0/G1 phase with concomitant reduction of cells in S phase **B.** Levels of Ki67, caspase-3, cleaved-PARP and GFAP in primary glioma cells in 3D and 2D cultures as measured using flow cytometry **C.** Results are shown as the means ± SD. **P*<0.05.

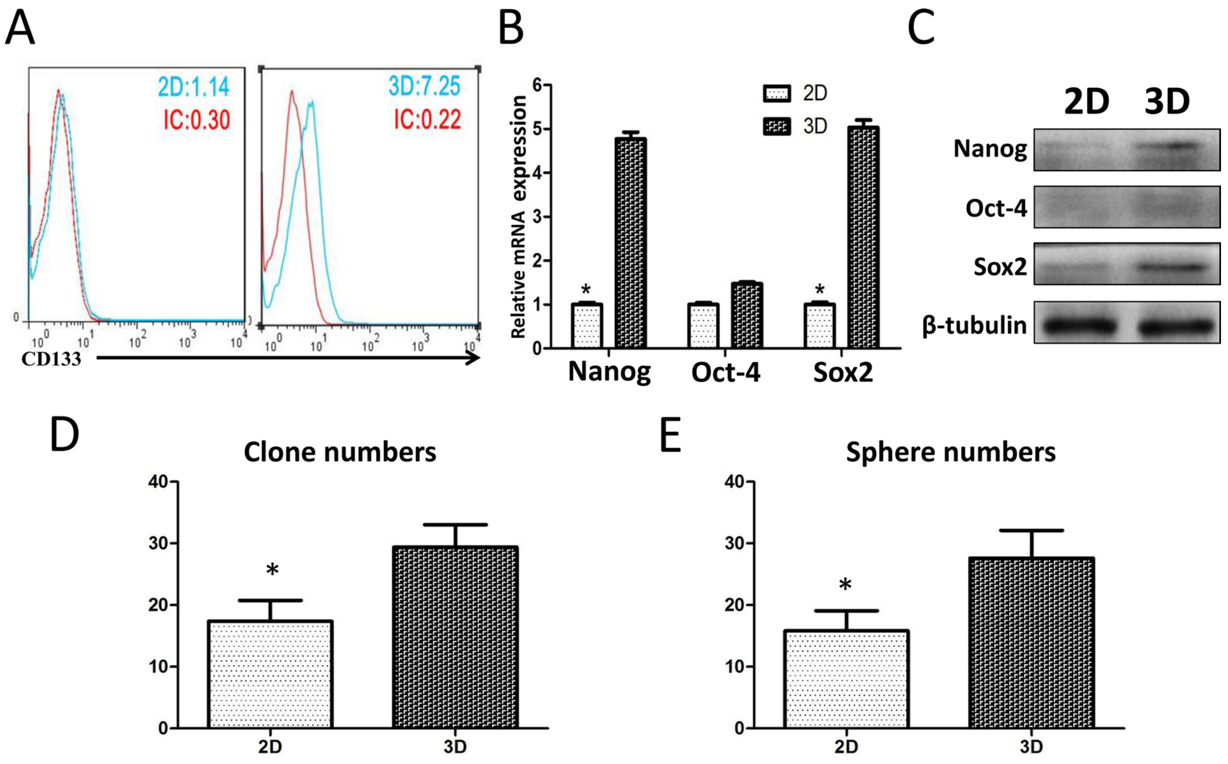

Supplementary Figure S3: Primary glioma cell stemness in 3D culture. Flow cytometry indicating CD133 expression **A.** Relative Nanog, Oct-4 and Sox2 mRNA **B.** and protein **C.** levels in primary glioma cells as determined by qRT-PCR and Western blotting. qRT-PCR data was normalized against GAPDH. Colony and sphere formation by primary glioma cells in 3D culture **D** and **E.** Results are shown as the means ± SD. *P<0.05.
